# Hyaluronan Accelerates Intestinal Mucosal Healing through Interaction with TSG-6

**DOI:** 10.3390/cells8091074

**Published:** 2019-09-12

**Authors:** Giusy Sammarco, Mohammad Shalaby, Sudharshan Elangovan, Luciana Petti, Giulia Roda, Silvia Restelli, Vincenzo Arena, Federica Ungaro, Gionata Fiorino, Anthony J. Day, Silvia D’Alessio, Stefania Vetrano

**Affiliations:** 1 IBD Center, Laboratory of Gastrointestinal Immunopathology, Humanitas Clinical and Research Center-IRCCS, Rozzano, 20089 Milan, Italy; giusy.sammarco@humanitasresearch.it (G.S.); mohammadkhaled.shalaby@st.hunimed.eu (M.S.); luciana.petti@live.com (L.P.); giuliaroda@gmail.com (G.R.); silvia.restelli@humanitasresearch.it (S.R.); Federica.Ungaro@humanitasresearch.it (F.U.); silvia.dalessio@hunimed.eu (S.D.); 2 Genomics Division, Wipro Life Sciences laboratory, WIPRO Limited, Bengaluru 560035, Karnataka, India; esudharshan@gmail.com; 3 Area of Pathology, Department of Woman and Child Health and Public Health, Fondazione Policlinico Universitario A. Gemelli-IRCCS, 00147 Rome, Italy; vincenzo.arena@policlinicogemelli.it; 4 Department of Biomedical Sciences, Humanitas University, Pieve Emanuele, 20090 Milan, Italy; gionataf@gmail.com; 5 Wellcome Trust Centre for Cell-Matrix Research, Faculty of Biology, Medicine, & Health, University of Manchester, Manchester Academic Health Science Centre, Manchester M13 9PT, UK; Anthony.Day@manchester.ac.uk; 6 Lydia Becker Institute of Immunology and Inflammation, Faculty of Biology, Medicine, & Health, University of Manchester, Manchester Academic Health Science Centre, Manchester M13 9PT, UK

**Keywords:** mucosal healing, epithelial regeneration, TSG-6, inflammatory bowel disease, experimental colitis, hyaluronan

## Abstract

Hyaluronan (HA) has proven to be beneficial in the treatment of several diseases. Recently, it has been shown that the local application of HA (IBD98E) improves endoscopic and clinical outcomes in subjects with active distal ulcerative colitis (UC). However, the mechanisms by which this polysaccharide exerts its beneficial effects are unclear. Here, we demonstrated that HA treatment in vitro and in vivo improved mucosal healing by accelerating intestinal epithelial regeneration. Indeed, mice treated with HA showed a faster recovery from colitis and reduced endoscopic signs of mucosal inflammation compared to those receiving saline. Furthermore, histological analysis revealed less ulcerated mucosa in mice treated with HA, characterized by re-epithelialized areas. TSG-6, the secreted product of TNF-stimulated gene-6, is an HA-binding protein shown previously to have tissue-protective properties and promote wound healing. Mucosal levels of TSG-6 increased in UC patients compared to the healthy controls and also after wounding in mice. TSG-6 deletion prevented the beneficial properties of HA in mucosal wound repair, suggesting that the interaction of HA with TSG-6 is crucial for intestinal epithelial regeneration. Overall these results are consistent with HA having a therapeutic effect via the promotion of mucosal healing in patients with ulcerative colitis.

## 1. Introduction 

Hyaluronan (HA) is a nonsulfated unbranched polysaccharide composed entirely of a repeating disaccharide of glucuronic acid (GlcA) and N-acetyl-glucosamine (GlcNAc) [1]. HA is synthesized by HAS enzymes (HAS1, HAS2, and HAS3) [2] as a single long-chain polymer reaching sizes up to ~10 MDa, and has a broad range of biological activities in mammalian tissues [3]. It is a ubiquitous component of the extracellular matrix (ECM), with a key role in tissue homeostasis, and is required for development, reproductive biology, and immune functions, exerting both structural and signaling activities [4,5]. The control of HA synthesis is, therefore, critical in ECM and cell biology. Under normal physiological conditions, high molecular weight hyaluronan (HMW-HA) suppresses the immune response acting as an anti-inflammatory molecule, acts as an anti-angiogenic factor, and exerts numerous receptor-mediated effects on cell adhesion, migration, mitosis, and inflammation [6]. The HA receptor CD44, which is expressed by a wide range of cell types (including stromal and immune cells), mediates many, but not all, of HA’s biological activities [7]. HA also has an important role in regulating the hydration of tissues (and their turgor), in part through its organization and retention of chondroitin sulfate proteoglycans [8]. Moreover, it is a component of the glycocalyx on epithelial cell surfaces that provide a protective barrier for tissues. However, upon tissue injury, HMW-HA is broken down by a family of enzymes called hyaluronidases, releasing low molecular weight HA fragments (LMW-HA). These species have been reported as having a wide range of effects, including, promoting tissue repair, stimulating the immune response, influencing tissue fibrosis, and acting as proangiogenic factors [8,9]. The molecular mechanisms by which HA exerts its physiological and pathological functions are still incompletely understood, but its interactions with specific HA-binding proteins are likely to be central to its diverse functions [8,10]. In biological solutions, such as synovial fluid, and in the vitreous of the eye, HA is largely present as a free polysaccharide without proteins attached. In this context, HMW-HA has important viscoelastic, sieving, and lubricating properties due to HA’s large domain size and the ability of each individual HA chain to bind to a huge number of water molecules. These properties, combined with the anti-inflammatory effects of HMW-HA, have proven to be beneficial in the treatment of articular and ocular diseases [11,12,13,14]. Moreover HA has been found to have potential for tissue remodeling and acceleration of wound repair and tissue restoration [15]. Recently, we have shown that the local application of a new formulation of a sodium hyaluronate gel (IBD98E) improves endoscopic and clinical outcomes in subjects with active distal Ulcerative Colitis (UC) [16].

Ulcerative colitis (UC) is a chronic, relapsing, remitting, and inflammatory disorder that affects millions of people around the world, with a rising trend [17]. It is one of the major forms of inflammatory bowel disease that leads to disabling clinical manifestations and enhances the risk of colectomy and colorectal cancer development [18]. The disease mainly affects the rectum and extends proximally for variable distances but does not involve the gastrointestinal tract proximal to the colon. The clinical course of UC is highly heterogeneous and ranges from a single episode to a potentially life-threatening continuous disease. Conventional medical treatment of UC includes the use of aminosalicylates, corticosteroids, and immunosuppressive drugs. However, none of the currently available therapies are effective for all patients [19]. In the active phase of the disease, the colonic mucosa presents diffuse ulcerations and epithelial necrosis, depletion of mucin from goblet cells, and a polymorphonuclear and lymphocytic infiltration involving the superficial layers of the colon to the muscularis mucosa. Consequentially, the structure and function of the mucosal area affected by the disease are compromised. The fundamental goal of therapy is, therefore, to achieve mucosal healing, and this relies on arresting mucosal bleeding, promoting epithelial regeneration, and restoring mucosal integrity [19]. The local administration of IBD98E in mild to moderate distal UC patients is a promising approach to promote endoscopic and clinical remission [16]. Although hyaluronan function is often dependent upon its interaction with proteins present on the cell surface and/or secreted into ECM, the molecular mechanisms by which HA promotes mucosal healing remains unclear. During inflammation, HA can become modified via the covalent transfer of heavy chains (HC) from the inter-α-inhibitor (IαI) to form HC-HA complexes, a process which is catalyzed by TSG-6 (the secreted product of TNF-stimulated gene-6 and also known as TNFα-induced protein-6 (TNFAIP6)) [20,21,22]. TSG-6-mediated production of HC-HA has been shown to be important during inflammation [23,24,25], ovulation [20,22,23,26] and organ morphogenesis [27], where, for example, some compositions of HC-HA have anti-inflammatory, anti-angiogenic and anti-fibrotic effects [23,25]. However, HC-HA complexes have also been associated with lung pathologies [23,28,29]. Furthermore, TSG-6 can modulate HA’s structure (in the absence of IαI) by directly crosslinking HA chains [30], thereby altering the hydrodynamic properties of HA and also enhancing the binding of HA to CD44 [30,31,32], which likely mediates some of TSG-6′s anti-inflammatory signaling effects [23,33]. TSG-6 also interacts with a large number of ligands in addition to HA (including chemokines) [34] and has been found to have therapeutic potential in a broad range of disease models [23]. Recently, we demonstrated that TSG-6 is crucial for the stemness properties of mesenchymal stem cells [35] and for their anti-inflammatory activities in the treatment of colitis [36]. Indeed, exogenous administration of TSG-6 ameliorated colitis and improved mucosal healing [36]. In this study, we provide evidence that local treatment with HMW-HA accelerates intestinal epithelial regeneration in a TSG-6-dependent manner.

## 2. Methods

### 2.1. Animals

C57BL/6N or BALB/c mice (six-eight weeks old female mice) were purchased from Charles River Laboratory (Calco, Italy), whereas Balb/c heterozygous TSG-6-deficient mice (C.129S6-Tnfaip6tm1Cful/J) were purchased from The Jackson Laboratory (France). Homozygous TSG-6-deficient mice, generated in Humanitas Clinical and Research Center, and wild-type littermates mice (six–eight weeks old), were used in this study. 

Mice were housed at the specific-pathogen-free (SPF) animal house of Humanitas Clinical and Research Center (Italy). All the procedures conformed to the principles of laboratory animal care, in compliance with national (Direttiva 2010/63/UE) laws and policies and approved by the Italian Ministry of Health (procedure N° 109/2012 authorized in 14/05/2012).

### 2.2. Patients 

Fourteen UC subjects, 7 men and 7 women (aged 35 to 65), with active mucosal disease defined by an endoscopic Mayo score ≥2 and a Riley Index ≥3, and 11 patients enrolled as healthy controls in this study, underwent colorectal cancer screening. Endoscopic mucosa biopsies were collected from all patients after informed consent was obtained. This study was approved by the ethical committee of the Humanitas Clinical and Research Center and conducted following national and international guidelines.

### 2.3. Experimental DSS-Induced Colitis

Acute colitis was induced by the addition of 3% dextran sulfate sodium (DSS) (MP Biomedicals, Illkirch, Europe) to drinking water and given ad libitum for 5 or 10 days. During the treatment, clinical parameters including body weight, bleeding, and stool consistency were recorded daily to calculate the disease activity index [37]. Hyaluronan (350–1800 MW) (IBD98E from MDT, Geneva, Switzerland) was administrated locally via enema (1 mg/mL) every other day after a gentle abdominal massage to release stool. 

### 2.4. Endoscopic Assessment

Mucosal damage was evaluated endoscopically before and after DSS-induced colitis and during HA treatment using the Coloview system under anesthesia [38]. The severity of colitis was assessed as a score from 0 to 15 points based on the evaluation of colon translucency (0–3 points), the presence of fibrin attached to the bowel wall (0–3 points), the granular aspect of the mucosa (0–3 points), the morphology of the vasculature (0–3 points), and the presence of loose stools (0–3 points).

### 2.5. Histological Analysis

At the end of the experiment, the colons were excised, measured, and fixed in 4% (*v*/*v*) formalin. After 24 h in the fixative, they were embedded in paraffin and sectioned at 2 μm for hematoxylin and eosin staining. The sections were assessed blinded (by a pathologist) according to their Rachmilewitz Scores [39]. Colonic damage was based on the presence/number of infiltrating inflammatory cells (0 (absent), 1 (low), 2 (mild), 3 (modest), and 4 (severe)); the presence of ulceration (no ulcer (0), mucosa (1), mucosa/submucosa (2), muscle/serosa (3), and whole epithelium (4)); and on the extent of the ulcer (no ulcer (0), punctate (1), low (2), moderate (3), and widespread (4)).

### 2.6. In Vivo Wound Healing

A mucosal wound was created by inserting flexible biopsy forceps with a diameter of 1 mm (French gauge 3). A discrete full-thickness biopsy was taken in the mucosal lining of the distal colon from each mouse before the start of HA-based therapy. This procedure was performed under anesthesia by using a cocktail of Ketamine (100 mg/Kg) and Xylazine (20 mg/Kg), administered into the muscle in a volume of 100 µL/mouse; 1 mg/mL HA (800–1200 MW) from IBSA (Leuven, Belgium) was administrated via enema (after a gentle abdominal massage to release stool) once a day for 3 days.

### 2.7. Immunohistochemical Staining

Serial paraffin sections (2 µm) of the distal colon were deparaffinized, hydrated, and blocked for endogenous peroxidase with 3% (*v*/*v*) H_2_O_2_ and then with Rodent Block M (BioCare, Concord, CA, USA) to prevent non-specific antibody binding. A primary antibody against Ki67 (1:800 dilution, rabbit anti-mouse; Cell Signaling Technology, Danvers, MA, USA) was incubated with the sections for 1 h at room temperature (RT). After this time, the sections were washed several times in 50 mM Tris-buffered saline (TBS, pH 7.4) and then incubated with a secondary goat anti-rabbit biotinylated antibody (Polymer Detection Kit; BioCare, Concord, CA, USA) for 1h at RT, followed by 30 min of incubation with a Streptavidin-Hoseradish Peroxidase conjuagte (BioCare, Concord, CA, USA). Antigen detection was obtained after 5 min of incubation with 3,3′-diaminobenzidine (BioCare, Concord, CA, USA). Slides were counterstained in Hematoxylin (DAKO; 1:5 in water), dehydrating through 3 changes of alcohol (70%, 90%, 100%) and cleared in 2 changes of xylene. Sections were mounted with medium EUKITT (Sigma-Aldrich, Darmstadt, Germany)). Images (122.943 pixels) were acquired (objective lens 20×/0.75; Olympus, Tokyo, Japan) with the DotSlide system (Olympus Soft Imaging Solution, Tokyo, Japan). Semi quantitative analysis of Ki67 expression was performed blind (by a pathologist).

### 2.8. Cell Culture

Caco-2 cells are a human colon cancer cell line that possess many morphological and functional similarities to human colonic epithelial cells. Caco-2 cells between a 23–35 passage were cultured in Dulbecco’s modified Eagle medium (DMEM; Gibco) supplemented with 10% (*v*/*v*) fetal bovine serum, 1 mmol/L l-glutamine, 1mmol/L sodium pyruvate, 0.1 mmol/L nonessential amino acids, and 100 U/mL) antibiotics (penicillin and streptomycin) at 37 °C in 5% CO_2_.

### 2.9. In Vitro Cytotoxicity

The cell viability of the Caco-2 cell was assessed by the reduction of 2,3-bis (2-methoxy-4-nitro-5-sulfophenyl)-2H-tetrazolium-5-carboxanilide inner salt (XTT) into an orange colored formazan product by the mitochondrial respiratory enzymes using a commercially available kit (AppliChem GmbH) according to the manufacturer’s instructions. To this purpose, 1.5 × 10^4^ of Caco-2 were plated into individual wells of tissue-culture microtiter plates with 96 wells. Following an incubation period of 18 h with the complete culture medium, the cells were washed with phosphate-buffered saline (PBS), pH 7.4, and subsequently treated with 0.1 mL of HA (0.5 or 50 µg/mL), or 20% (*v*/*v*) dimethyl sulfoxide (DMSO) as the negative control or medium alone as the positive control, for 24 h at 37 °C in 5% CO_2_. After 24 h of incubation, the cells were analyzed by microscopy for any morphological changes and then treated with 50 µL of XTT reaction solution (added to each well). After an additional 24 h of incubation, the plates were analyzed at a wavelength of 540 nm using a spectrophotometer. The absorbance values were normalized to the values of absorbance of the complete medium without the addition of DMSO and reported as a % of mitochondrial activity.

### 2.10. Cell Proliferation Assay

Caco-2 cell proliferation was evaluated in the presence of HA (0.5 µg/mL or 50 µg/mL) by the nucleotide 5-Bromo-2′-deoxyuridine (BrdU) incorporation test (AppliChem GmbH), a quantitative colorimetric assay based on the ability of BrdU to bind DNA during the replication phase. Next, 5 × 10^4^ Caco-2 cells were plated in 96 well plates and incubated with 100 ng/mL Epidermal Growth Factor (EGF) in DMEM (as positive control) without fetal bovine serum (as negative control) and in the presence of two different concentrations of HA for 24 h at 37 °C in a humid atmosphere. After this time, 10 µM BrdU was added to the cells for 2 additional hours. At the end of the incubation, the medium was removed, and the cells were fixed with 200 µL of FixDenat solution. After a further incubation period of 30 min at RT, the FixDenat solution was removed and replaced by 100 µL of anti-BrdU antibody labeled with peroxidase (anti-BrdU POD) (1:100) provided in the kit. After 90 min, the cells were first washed 3 times with 300 µL of PBS and then incubated with 100 µL of the substrate, provided in the kit, for 5–10 min, at RT. Subsequently, the absorbance values were acquired, and the results were reported as optical density.

### 2.11. In Vitro Wound Healing

Wound healing in response to HA stimulation was assessed by a scratch assay on a Caco-2 cell monolayer as previously reported [40]. Briefly, 5 × 10^4^ Caco-2 cells were seeded into each well of culture inserts and incubated at 37 °C in a humidified atmosphere with 5% CO_2_. After 24 h, the culture inserts were gently removed using sterile tweezers, and a scratch was made in the monolayers. Then, the cells were incubated for 24 h with HA at different concentrations (0.5 or 50 µg/mL), EGF (100 ng/mL), or medium alone. To evaluate the cells’ migratory capacity separately from cell proliferation, the cells were pre-incubated with or without mitomycin C (30 µg/mL) (Sigma-Aldrich) for 2 h at 37 °C. Photographs of the wounded area were taken immediately before stimulation (0 h time point) and after 24 h to monitor the closure of the wounded area. The percentage of wound closure was calculated by ImageJ software as (Area initial − Area final)/Area initial × 100.

### 2.12. RNA Extraction

Total RNA was extracted from human and mouse biopsies using the RNeasy Lipid Tissue Mini kit (*Qiagen*), according to the manufacturer’s instructions. Primer sequences used are reported in Table 1. Glyceraldehyde-3-phosphate dehydrogenase (GAPDH) was used as a PCR control. The relative abundance was expressed as 2^−Δ*Ct*^.

The level of TSG-6 was quantified in Caco-2 cells by quantitative real-time polymerase chain reaction (qPCR). Here, the total RNA was extracted from Caco-2 cells using the Qiagen RNeasy Mini Kit (Qiagen), and the qPCR reaction then was performed on synthesized complementary DNA using a SYBR Green PCR Master Mix (Applied Biosystem).

### 2.13. Statistical Analysis

Data were analyzed using Graphpad software (Prism 8 for OS X, version 8.1.2, San Diego, CA, USA) and expressed as the mean ± SEM. An unpaired Student’s *t*-test and Mann-Whitney U test were used for comparison of two groups, whereas a one-way ANOVA with Bonferroni post-test analysis was used for the comparison of multiple groups. Categorical variables were compared using Pearson’s chi-squared test. Statistical significance was set at *p* < 0.05.

## 3. Results

### 3.1. HA Accelerates the Recovery Phase after Colitis Insult

**Figure 1:**
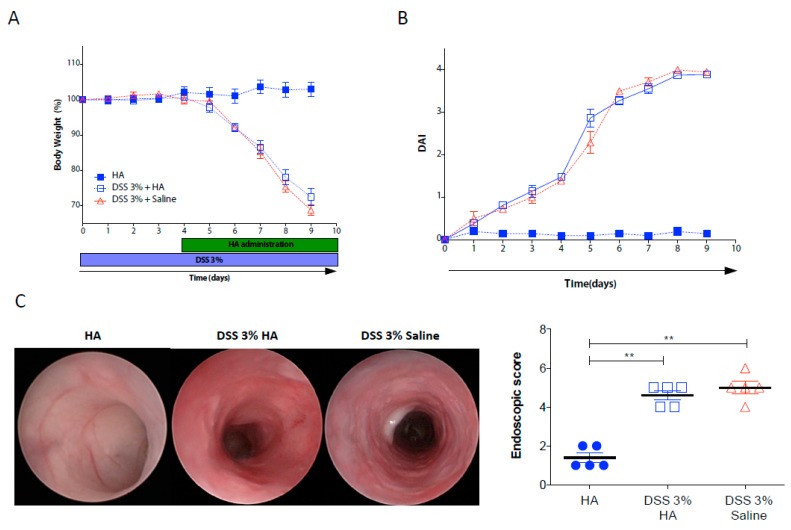
Hyaluronan (HA) treatment did not ameliorate the acute phase of experimental colitis. (**A**,**B**) Body weight and disease activity index (DAI) monitored over 9 days of dextran sulfate sodium (DSS)-induced colitis in C57BL/6N wild type mice receiving applications of high molecular weight HA (1mg/mL) or saline every other day via enema, commencing from day 4. Healthy mice receiving only HA served as controls. **C** Representative endoscopic images of the intestinal mucosa and endoscopic score (summarising the mucosal damage) at day 9 of DSS administration. Data represent the mean values ± SEM (*n* = 5/group); ** *p* < 0.01 compared to control group by one-way-ANOVA.

To shed light on the therapeutic mechanisms of local administration of HA in inflamed mucosa, we induced colitis in wild type mice by administration of 3% DSS for 9 days. Starting from day 4 after DSS administration, when inflammation was generally established, we divided the mice into two groups: one receiving HA (1 mg/mL), and the second one receiving only saline as a control, every other day. Daily, we monitored the clinical parameters during the entire experiment. No differences in terms of body weight changes were observed between the two groups of colitic mice (Figure 1A). Both the HA- and saline-treated groups showed a comparable percentage of weight loss (over 30% at day 9), although mice receiving HA displayed a slight improvement (but not significant) compared to the saline group at day 8 and 9. Similarly, the Disease Activity Index (DAI), calculated based on weight changes, bleeding, and the consistency of stools, which reflects the severity of the colitis, did not reveal significant differences between the HA and saline groups (Figure 1B). Indeed, the endoscopic examination at day 9 showed similar lesion characteristics of mucosal damage after colitis induction (e.g., erosions, thickening of the colon, and granularity) between the two groups (Figure 1C,D), thereby indicating that HA treatment does not exert therapeutic effects in the acute phase of severe colitis.

An efficient resolution of inflammation is one of the most important aspects to which the latest therapies for the treatment of UC patients aspire. Indeed, to maintain long-term remission in patients with UC, the main goal is to achieve mucosal healing, which relies on arresting mucosal bleeding and promoting epithelial regeneration/integrity. Therefore, we investigated the effects of HA on the recovery phase following the DSS challenge.

**Figure 2:**
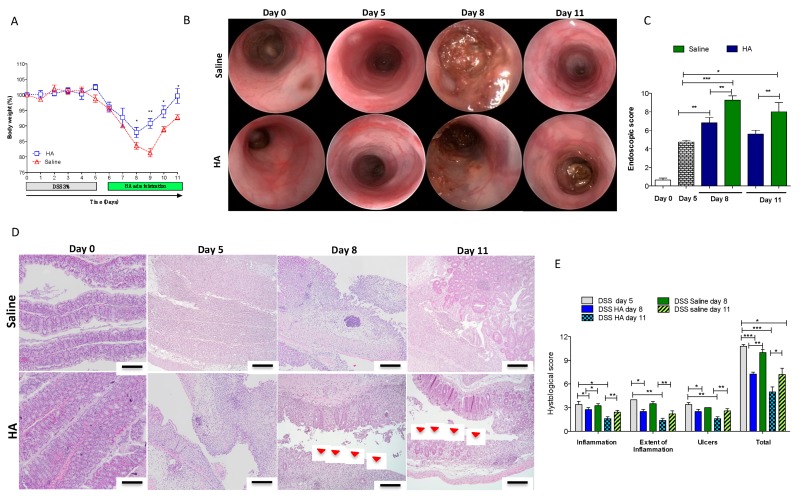
HA treatment accelerated the recovery phase of acute colitis. Acute colitis was induced in C57BL/6N wild type mice by 3% DSS administration in drinking water ad libitum for 5 days. After this time, the DSS was replaced with regular water, and, starting on day 6, mice received daily local applications of high molecular weight HA (1mg/mL) or saline via enema. (**A**) Body weights monitored over the entire experiment. (**B**–**C**) Representative endoscopic images of the intestinal mucosa and endoscopic score, summarising the mucosal damage at day 0 (before DSS administration), at day 5 after DSS administration, and at day 8 and 11 (after 3 and 5 days, respectively, of HA or saline treatment). (**D**–**E**) Paraffin sections of colon were stained with HE to evaluate histological changes and to grade mucosal inflammation at day 0, 5, 8, and 11, based on the Rachmilewitz score. Representative histological images (**D**) and histograms of quantitative analysis of mucosal damage (**E**) are provided for all groups of mice. Red arrows indicate sites of epithelial regeneration. The sections were photographed at 20x magnification. Data represent the means ± SEM (*n* = 5/group); * *p* < 0.05; ** *p* < 0.01; *** *p* < 0.001 compared to control group by one-way-ANOVA.

To this end, we challenged mice with 3% DSS for only 5 days, an exposure time sufficient to promote mild–moderate colitis characterized by weight loss (over 15%) and epithelial injury with mucosal bleeding (Figure 2A and Figure S1A). After 5 days, we replaced regular drinking water, and on day 6, we started HA treatment (1 mg/mL) or saline every other day via enema. Interestingly, HA administration led to a slight recovery of weight changes soon after the first application and achieving a significant amelioration after two days of application (day 8), and immediately after the second injection (day 9) (Figure 2A). The rapid weight gain correlated with a reduced bleeding score (*p* < 0.05) (Figure S1A) and with an amelioration of mucosal damage, as recorded by the reduced endoscopic colitic signs/scores (Figure 2B,C). Indeed, at day 8, the mucosa of mice receiving HA showed mild endoscopic colitis grading with moderate thickening of the colon wall, moderate changes of the vascular pattern, and mild mucosal bleeding compared to mice receiving saline (which, on the contrary, displayed marked granularity of the mucosal surface, marked thickening of the colon wall, and mucosal erosions). At the end of the experiment (day 11), after three HA applications, the HA-treated mice recovered 100% of their initial weight compared to 90% of those receiving saline (Figure 2A). Despite not being significant, they also showed slight improvement in colon length (Figure S1B) and exhibited reduced endoscopic signs of mucosal inflammation (Figure 2B–C) (*p* < 0.01). Furthermore, histological analysis revealed reduced ulcerated areas in mice treated with HA compared to saline controls (Figure 2D) (*p* < 0.01) at the day 8 and day 11 time points. The analysis based on the Rachmilewitz score revealed a reduction of inflammation and the extent of the inflammation and ulceration, characterized by larger areas re-epithelialized in HA treated mice compared to the colonic mucosa of mice treated with saline (Figure 2E) (*p* < 0.05). In order to address the role of HA in preventing intestinal inflammation, we pre-treated mice every other day with HA (1 mg/mL) via enema 7 days before inducing acute colitis under the same experimental conditions as in the previously described experiments (see above). However, the pre-treatment with HA did not exert protective effects on the clinical parameters (body changes, DAI, and histological analysis) in colitic mice (Figure S2A–C).

### 3.2. HA Treatment Promoted Epithelial Cell Proliferation

**Figure 3:**
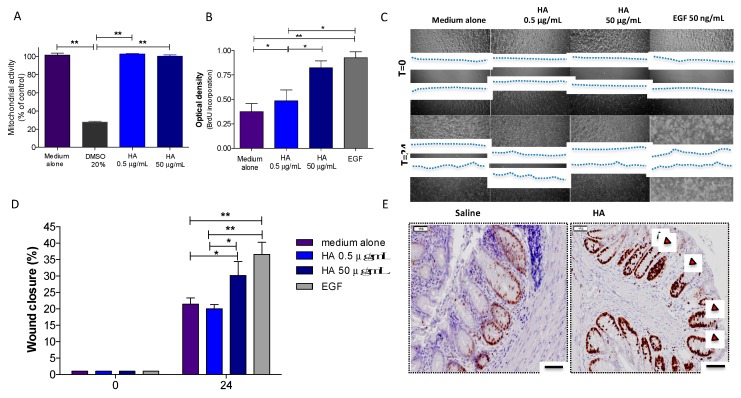
HA treatment promoted epithelial cell proliferation. (**A**) Effect of HA on the viability of Caco-2 cells determined by using the 2,3-bis (2-methoxy-4-nitro-5-sulfophenyl)-2H-tetrazolium-5-carboxanilide inner salt assay. Cells were treated with two different doses of HA (0.5 and 50 μg/mL) or 20% DMSO (as negative control) and incubated for an additional 24 h. (**B**). The effect of HA on cell proliferation measured by a BrdU cell proliferation assay. Cells were treated with two different doses of HA (0.5 and 50 μg/mL) or epidermal growth factor (EGF) (50 ng/mL, as positive control) and incubated for 24 h. (**C**,**D**). Representative images and quantitation of the wound closure in Caco-2 cells after incubation with HA (0.5 and 50 μg/mL) or EGF (50 ng/mL, as positive control) for 24 h. Bars represent the mean ± SEM of a single experiment assayed in triplicate and are representative of the three separate experiments. * *p* < 0.05 and ** *p* < 0.01 compared to medium alone. The cell monolayers were photographed at 10x, and the dotted blue lines show the extent of the remaining wound area. (**E**). Paraffin sections of colon from mice receiving HA (1mg/mL) or saline for 72 h after mucosal wounding. The sections were stained for the Ki67 marker (brown signal). Red arrows indicate the proliferation of epithelial cells along the crypt axis. The sections were photographed at 20×; (*n* = 5/group); Bar = 50 μm.

Epithelial repair/restitution represents a crucial aspect of the resolution of intestinal inflammation. Several pieces of evidence support HA as a key component of the extracellular matrix involved in different biological process, such as proliferation and migration. To address the hypothesis that HA plays a role in the regenerative process of the intestinal mucosa, we assessed its effects on proliferation in vitro using Caco-2 cells. Firstly, we measured the viability of Caco-2 cells exposed to HA at two different concentrations (0.5 and 50 µg/mL). After 24 h of exposure, Caco-2 cells showed 100% enzymatic mitochondrial activity relative to the medium alone (Figure 3A), thereby indicating no cytotoxic effects of HA on the viability of colonic epithelial cells. This was verified by also stimulating the cells in presence of 20% DMSO, which is toxic for the cells and markedly compromised their mitochondrial activity (Figure 3A). Secondly, we explored the effects of HA on epithelial cell proliferation by analyzing BrdU incorporation. To do this, Caco-2 cells were plated for 24 h in the presence of different concentrations of HA (0.5 and 50 µg/mL), followed by 30 min of incubation with BrdU before detection with an HRP-conjugated secondary antibody. At a concentration of 50 µg/mL, HA promoted significant epithelial cell proliferation compared to the medium alone (*p* < 0.05), similar to EGF (50 ng/mL) (*p* < 0.01) used as positive control, whereas at 0.5 µg/mL, HA did not show any proliferative properties (Figure 3B), indicating that HA promotes epithelial regeneration in a dose-dependent manner.

Similar results were observed when we measured the in vitro capacity of HA to promote wound healing by scratch wounding a confluent monolayer of Caco-2 cells. HA at a concentration of 50 µg/mL accelerated Caco-2 wound closure after 24 h by 30.1% (*P* < 0.05 vs. medium alone and *P* < 0.05 vs. HA 0.5 µg/mL), which is comparable to EGF (36.0% acceleration; *P*< 0.01 vs. medium alone and *P* < 0.01 vs. HA 0.5 µg/mL) (Figure 3C,D). Interestingly, no wound closure was found in response to any concentrations of HA after pre-treatment with mitomycin C, indicating that HA promotes proliferation but does not affect the migration of epithelial cells (data not shown).

To validate the in vitro assays, we investigated the effects of HA on mucosal healing by stimulating the regeneration of epithelial cells in vivo. To this end, we created a mucosal wound by taking a discrete biopsy from the distal colon of mice and treated them daily with HA (1 mg/mL) for 72 h. The immunostaining for Ki67, a marker of proliferation, revealed augmented epithelial cell proliferation surrounding the area of the wound after HA treatment (Figure 3E). HA significantly contributed to the proliferation of stem cells at the base of the crypt and their migration upward along the crypt axis, thereby favoring epithelial cell turnover. Overall, these results indicate a strong capacity of HA to promote intestinal epithelial regeneration.

### 3.3. Inflammatory Conditions Modulated the Synthesis of HA and the Expression of CD44 Receptor

**Figure 4:**
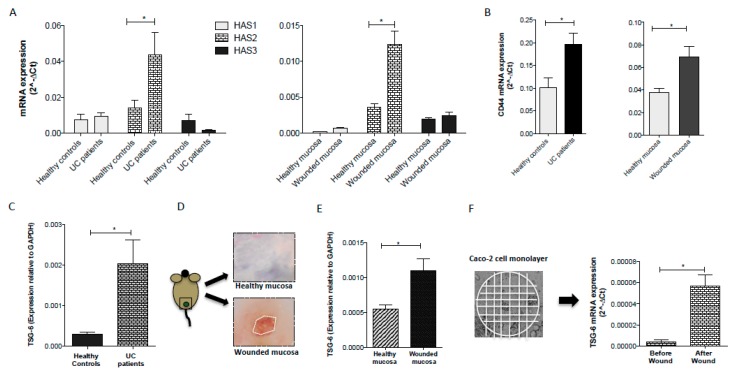
Inflammation and tissue damage induced synthesis of HA and the expression of CD44 and TSG-6. Gene expression of (**A**) Hyaluronan Synthase 1-3 (HAS1-3), (**B**) CD44, and (**C**–**E**) TSG-6 in UC patients vs. healthy controls (left panel) and in the mucosal wound of wild type mice (right panel). A mucosal wound was created by taking a discrete biopsy from the distal colon of mice. After 24 h, the wounded area was collected and analyzed. (**D**) Representative image of a wound site (white dotted line indicates wounded area) was acquired under a stereomicroscope at 4×. (**F**) Confluent Caco-2 cells, plated in a 6-well plate, were scratched in several horizontal and vertical straight lines with a p200 pipette tip. After 24 h at 37 °C, the cells were collected and processed for gene expression analysis of TSG-6. For all qRT-PCR results, data reflect the mean ± SEM from three independent experiments. The results were normalized to glyceraldehyde-3-phosphate dehydrogenase (GAPDH) mRNA. * *P* < 0.05 by one-way ANOVA. (*n* = 14 UC patients and 11 healthy controls); (*n* = 10 animals/group).

HA is synthesized by hyaluronan synthases, a class of integral membrane proteins present as three isoforms called HAS1, HAS2, and HAS3 [2]. The expression and activity of HAS1, HAS2 and HAS3 are induced by pro-inflammatory factors [41,42]. Indeed, HA synthesis is greatly increased in inflammatory conditions [43]. In line with these previous observations, we found a significant (*p* < 0.05) up-regulation of HAS2 gene expression in the mucosa of active UC patients compared to the mucosa of the healthy controls (Figure 4A; left panel) and in the intestinal mucosa of mice 24 h after wounding the distal colon (Figure 4A; right panel). No significant differences were observed in the levels of HAS1 and HAS3 between healthy and inflamed/damaged mucosa in humans and mice (Figure 4A). Many biological properties of HA are mediated by its interaction with the cell surface receptors that activate distinct downstream signaling [7]. These include CD44, which is widely expressed and implicated in mediating a broad range of HA functions, especially in the context of inflammation. To assess the role of CD44 in the intestinal inflammatory response, we analyzed the mucosal gene expression of CD44 in UC patients and our murine model of mucosal wound injury. Interestingly, in both human and murine samples, the levels of CD44 were highest in the inflamed/wounded mucosa compared to the healthy mucosa (Figure 4B; left panel and right panel respectively; *p* < 0.05). These data correlated with the augmented synthesis of HA.

### 3.4. TSG-6 Expression is Upregulated following Intestinal Injury

There is clear evidence that TSG-6 can modulate the interaction of HA with CD44 and, thus, might regulate HA signaling at sites of inflammation [26,36,37,38]. Recently, we demonstrated that TSG-6 is crucial for the anti-inflammatory properties exerted by mesenchymal stem cells in the treatment of colitis and that the administration of TSG-6 ameliorates colitis and improves mucosal healing [36]. However, whether the properties of HA that promote epithelial cell regeneration are mediated by its interaction with TSG-6 remain to be investigated. TSG-6 is not constitutively expressed in most adult tissues (brain, lung, and skin are exceptions to this), but its expression is induced in response to inflammatory conditions [23,26]. Here, we found that TSG-6 mRNA was present at low levels in the mucosa of healthy controls but with seven-fold higher expression in UC patients (Figure 4C). To explore the involvement of TSG-6 in the processes of mucosal healing, we created a mucosal wound in the distal colon of mice (Figure 4D). After 24 h, the wounded area was collected and analyzed for the expression of TSG-6. In line with the human data, the mRNA levels of TSG-6 resulted in a two-fold increase in the wounded mucosa vs. the healthy mucosa (Figure 4E) consistent with the involvement of TSG-6 in the process of wound repair [44,45,46,47,48]. While TSG-6 can be produced by mesenchymal stem/stromal cells in response to signals from injured tissue, it can also be secreted/released by a broad range of cell types during tissue injury/inflammation [23,26], including epithelial cells [49], which are the key players in the wound healing processes.

To explore whether TSG-6 plays a role in intestinal epithelial regeneration after injury, we analyzed the mRNA levels of TSG-6 in a monolayer of Caco-2 cells before and after inducing a wound. To this end, a cell monolayer was scratched in several horizontal and vertical straight lines with a p200 pipette tip (Figure 4F) and after repetitive washes to remove cell debris, fresh medium was replaced for 24 h at 37 °C. Then, the cells were collected and processed for total RNA extraction. Analysis of mRNA in the monolayer of Caco-2 cells showed very low levels of TSG-6 under steady-state conditions and a marked increased after wounding (Figure 4F). The modulation of TSG-6 expression in intestinal epithelial cells in this scratch assay raised the possibility that increased levels of TSG-6 could mediate the activities of HA described above, e.g., by modulating cell migration [50].

### 3.5. The Lack of TSG-6 Abolished the Therapeutic Effects of HA Treatment

**Figure 5:**
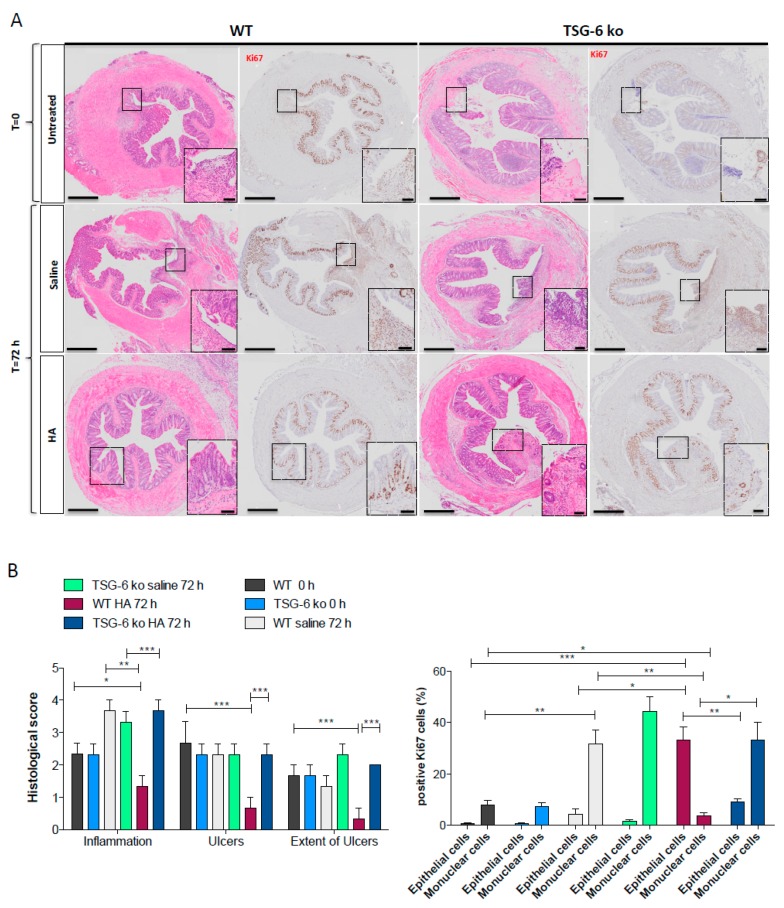
TSG-6 mediates HA-induced epithelial cell regeneration. (**A**). Representative photomicrographs of the intestinal mucosal wound created in the distal colon of TSG-6-deficient mice (TSG-6 ko) and their WT littermates (WT); 24 h after wounding the mice were treated daily with HA (1 mg/mL) or saline for 72 h via enema. Paraffin sections of colon were stained with HE and for the Ki67 marker to analyze the histopathological and proliferative changes during mucosal regeneration. The sections were photographed at 20× or 40× for insets (*n* = 5/group); Bars = 500 μm and 50 μm, respectively. (**B**) (left panel) Representative histograms of the quantitative histological analysis of mucosal damage in all groups of mice; (right panel) quantitative analysis of Ki67-positive cells expressed as percentage of epithelial cells or mononuclear cells in the mucosa of all groups of mice. Data represent the means ± SEM (*n* = 5/group); * p < 0.05; ** p < 0.01; *** p < 0.001 compared to control group by one-way-ANOVA.

To test this hypothesis, we created a mucosal wound by taking a discrete biopsy from the distal colon of TSG-6 deficient (TSG-6 ko) mice and their littermates (WT). After 24 h post wounding, mice were treated with HA (1 mg/mL) via enema every day for 72 h. HA treatment accelerated the tissue repair in WT mice (Figure 5A, panel left), confirming our previous results. Indeed, mice receiving HA showed, after 72 h, a complete epithelial regeneration compared to time 0 (when the wound was created) and compared to the saline-treated mice (*p* < 0.05). The Ki67 staining revealed, in fact, pronounced epithelial cell proliferation in HA-treated mice (consistent with mucosal healing), whereas those treated with saline displayed a marked proliferation of inflammatory cells without mucosal regeneration (Figure 5B). Conversely, TSG-6 ko mice either treated with HA or saline showed no mucosal healing. Indeed, at 72 h both HA- and saline-treated TSG-6 ko animals showed a massive infiltration of immune cells in the area of the wound without appreciable improvement in epithelial proliferation (Figure 5A (panel right TSG-6; ko); Figure 5B). This shows that the beneficial effects of HA were lost in the TSG-6-deficient mice. Interestingly, TSG-6 ko mice showed a significant reduction in the expression of HAS2 compared to WT mice 72 h after the wounding (Figure S3A). Indeed, we found a positive correlation between the expression of HAS2, TSG-6, and CD44 72 h after wounding in WT mice (Figure S3B). Overall, these data demonstrate that TSG-6 may play a role in mediating HA’s therapeutic benefits to mucosal wound healing.

## 4. Discussion

Ulcerative colitis is a chronic disease for which there is currently no curative therapy. The long-term response to drug treatment is less than 50%, and all existing drugs are associated with potential toxicity and systemic (side) effects [17,19]. Furthermore, at present, the long-term outcome for UC is unfavorable in many patients, with surgery rates up to 20–30% [19]. Therefore, there is a great need for new and cost-effective drugs with greater efficacy and tolerability, not only to ensure clinical remission but also to promote mucosal healing.

Mucosal healing is considered an important endpoint to assess therapeutic effects on inflammatory bowel disease, based on the lack of erythema and friability at endoscopy and on the absence of ulceration or erosions. In this present study, our data support the administration of HA as a new approach for promoting the intestinal re-epithelization and mucosal healing in the recovery phase of active ulcerative colitis, but not in the acute phase. The local application of HA accelerated the recovery of clinical parameters and improved tissue regeneration. Endoscopic and Rachmilewitz histological scoring revealed reduced bleeding and hyperemia, but also showed a decrease in ulcers, granularity, and erosions. These results reinforced our previous findings on the clinical remission and endoscopic healing in active UC patients (47.6%) after 28 daily applications of IBD98E, a medical device composed of HA at a molecular weight of 0.35–1.8 MDa [16]. This treatment was safe, and patients experienced no side effects.

However, the mechanisms by which HA promotes clinical and endoscopic remission in UC patients were, until now, unclear. Hyaluronan is an important component of the extracellular matrix, where the structure of this polysaccharide has been conserved throughout vertebrate evolution. As well as having a key role in tissue hydration, HA also regulates cellular functions. It is found at high concentrations in several soft connective tissues (e.g., skin), and contributes to many aspects of wound repair (including inflammatory and granulation stages), both promoting and limiting inflammation, as well as facilitating cell migration, proliferation, and angiogenesis. In this regard, HAS enzymes, which are responsible for HA biosynthesis, are crucial for the regulation of the wound healing process. We found that, in response to experimental mucosal injury, the mRNA levels of HAS2 drastically increased in the wounded area within 24 h of injury, but not those of HAS1 or HAS3. Similarly, in active UC patients, we observed a significant up-regulation of HAS2 compared to healthy controls. Although HAS enzymes are reported to be up-regulated upon inflammatory conditions in some contexts [51,52], we did not find any increase of the HAS1 and HAS3 isoforms in active UC. Further studies are necessary to address the reasons for this differential expression among the three isoforms, e.g., to determine whether their expression is of a cell type specific, and which inflammatory mediators induce HAS2 transcription in UC. Nevertheless, the increased expression of HAS2 likely leads to an increased synthesis of HA in active disease. In line with this finding, de la Motte et al. detected an accumulation of HA polymer in the mucosa of inflamed IBD [53]. 

In normal tissues, HA is synthesized as a long polymer with a high molecular weight (>1000 kDa) with a role in supporting ECM structure and hydration. Moreover, HMW-HA can suppress the inflammatory response [6,10] and prevent new blood vessels from forming [54,55], thereby leading to the perpetuation of inflammatory conditions [56]. 

Therefore, it is plausible that the increased HAS2 levels in UC patients is a physiological response of mucosa to synthesize HMW-HA to counteract the tissue damage and promote tissue repair; however, the newly synthesized HA is probably not sufficient to prevent an inflammatory response. Alternatively, HA might undergo degradation into lower molecular weight fragments, which could sustain inflammatory processes [57]. In support of this theory, we observed no acceleration in the recovery phase of colitic mice receiving LMW-HA (≤5 kDa) (data not shown), thus suggestive of an important role of HA size in this system. Based on this hypothesis, the exogenous applications of HMW-HA could represent an efficient approach to counteract the pro-inflammatory effects of HA fragments and accelerate mucosal regeneration. How different sizes of HA mediate their differential biological effects is not well understood at a molecular level. While it is possible that LMW-HA is recognized by distinct receptors (such as TLRs), there is no current evidence supporting a direct interaction. Another possibility is that LMW-HA competes for the interaction of HMW-HA with classical HA receptors (e.g., CD44), thus removing the anti-inflammatory and anti-angiogenic protection of HMW-HA. Importantly, such competition is feasible and can occur effectively due to the ‘superselective’ nature of HA-receptor interactions [8,58]. While several HA receptors have been identified, CD44 is probably the most relevant here since it regulates many biological functions, including cell interactions, migration, and proliferation. Although CD44 is ubiquitously expressed, its levels can be modulated during the injury response [7,59]. Indeed, we observed an increase in the expression of CD44 in the inflamed mucosa of UC patients and in the region of wound injury in mice. The binding affinity of HMW-HA to CD44 is influenced by the crosslinking of HA with proteins such as TSG-6 [30,31], an ~35-kDa secreted protein mediating many anti-inflammatory and tissue protective properties [23]. TSG-6 interacts with HA via its Link module domain [60] where this induces self-association of TSG-6 to form oligomers that crosslink HA chains [30], thereby altering the polysaccharide’s hydrodynamic properties. This crosslinking is thought to drive the clustering of CD44 and other HA receptors [8,23]. Furthermore, TSG-6 acts as a catalyst to transfer heavy chains (HCs) from the serum-derived proteoglycan IαI onto HA to form a covalent HC-HA complex that can also enhance the interaction of HA with CD44 [24,32]. 

As noted before, TSG-6 is not constitutively produced in most adult tissues, but it is highly upregulated in response to inflammatory conditions [23,26]. Interestingly, TSG-6 is expressed in normal skin (co-localized with HA and HCs) [61] and has been found to promote wound healing in a number of disease models [23,44,45,46,47,48]. We found low levels of constitutive TSG-6 mRNA in healthy mucosa with an increased expression of TSG-6 in UC patients and in mice following wounding. This increase was commensurate with increased levels of CD44, which suggests that CD44 and TSG-6 may co-operate to mediate the HA therapeutic activities seen in this study. Importantly, the treatment of HMW-HA failed to promote wound repair in the TSG-6 null mice. After 72 h of HA treatment, TSG-6-deficient mice showed incomplete tissue regeneration compared to WT mice along with massive infiltration of immune cells in the area of the wound without appreciable improvement in epithelial proliferation. Altogether, these data suggest that the beneficial effects of local applications of HMW-HA are perhaps mediated by the presence of TSG-6. This protein can be expressed (and secreted) upon injury/inflammation by a broad range of cell types [23,26], including epithelial cells [49,62]. Our in vitro data demonstrate that upon wounding intestinal epithelial cells, which are the key players in wound healing processes, these cells up-regulate the levels of TSG-6. It is reasonable to assume that in response to inflammation/injury, intestinal mucosa counteracts the inflammatory response and tissue damage by activating HMW HA-mediated signaling. In this scenario, epithelial cells increase the levels of TSG-6 to enhance the interaction of HMW HA with CD44, which in turn activates downstream signaling for regenerative processes. In this regard, there is evidence that the protective effects of TSG-6 can be mediated in a CD44-dependent manner [23,33,63]. Further studies are necessary to determine the molecular mechanisms whereby the interactions between TSG-6 and HA accelerate mucosal healing, and to address whether these effects are indeed mediated by the binding to CD44 or whether alternative pathways, such as TLR-dependent signaling, are involved [64]. Although our in vitro data show the crucial role of HA in promoting intestinal epithelial regeneration without affecting the migration of epithelial cells, additional in vivo studies are also needed to corroborate this aspect of the study (i.e., there remains a large difference in the HA concentrations used in in vivo versus in vitro experiments that could influence specific cell processes).

Overall, the local application of HMW HA represents a safe and efficient approach to ensure long-term mucosal healing in UC patients.

## Figures and Tables

**Table 1:** List of the primer sequence used in the study.

Gene	Primer Sequences
mm-HAS 1 h-HAS 1	Forward: 5′-GCGAGCACTCACGATCATCTT-3′ Reverse: 5′-GTCCATAGCGATCTGAAGCCA-3′ Forward: 5′-GAGCCTCTTCGCGTACCTG-3′ Reverse: 5′-CCTCCTGGTAGGCGGAGAT-3′
mm-HAS 2 h-HAS 2	Forward: 5′-GTACGGTGCCTTTTTAGCCTC-3′ Reverse: 5′-TAATCGGGGTTTCAAGGGACT-3′ Forward: 5′-TCCTGGATCTCATTCCTCAGC-3′ Reverse: 5′-TGCACTGAACACACCCAAAATA-3′
mm-HAS 3 h-HAS 3	Forward: 5′-CAATCGCCAGGAAGATACCTAC-3′ Reverse: 5′-GGAAATTGCTACGCCACACAA-3′ Forward: 5′-CAGCCTATGTGACGGGCTAC-3′ Reverse: 5′-CCTCCTGGTATGCGGCAAT-3′
mm-CD44 h-CD44	Forward: 5′-ACTTTGCCTCTTGCAGTTGAG-3′ Reverse: 5′-TTTCTCCACATGGAATACACCTG-3′ Forward: 5′-CTGCCGCTTTGCAGGTGTA-3′ Reverse: 5′-CATTGTGGGCAAGGTGCTATT-3′
mm-GAPDH h-GAPDH	Forward: 5′-CCATGTTCGTCATGGGTGTG-3′ Reverse: 5′-CAGGGGTGCTAAGCAGTTGG-3′ Forward: 5′-TGTGTCCGTCGTGGATCTGA-3′ Reverse: 5′-CCTGCTTCACCACCTTCTTGA-3′

mm = murine, h = human.

## Supplementary Materials

The following are available online at https://www.mdpi.com/2073-4409/8/9/1074/s1, Figure S1: Clinical parameters recorded during the recovery phase in colitic mice after the administration of hyaluronan. Figure S2: Lack of effects in preventing acute colitis in wild type mice receiving a pre-treatment with Hyaluronan. Figure S3: Analysis of mRNA levels of Hyaluronan-TSG-6/CD44 axis in wild type and TSG-6 deficient mice upon mucosal wounding.
