# Supplementary material for: Hyaluronan Accelerates Intestinal Mucosal Healing through Interaction with TSG-6
Source: Cells. 2019 Sep 12;8(9):1074. doi: 10.3390/cells8091074 (PMC6769700; doi:10.3390/cells8091074)
Supplement: Supplementary file 1 [file cells-08-01074-s001.pdf]

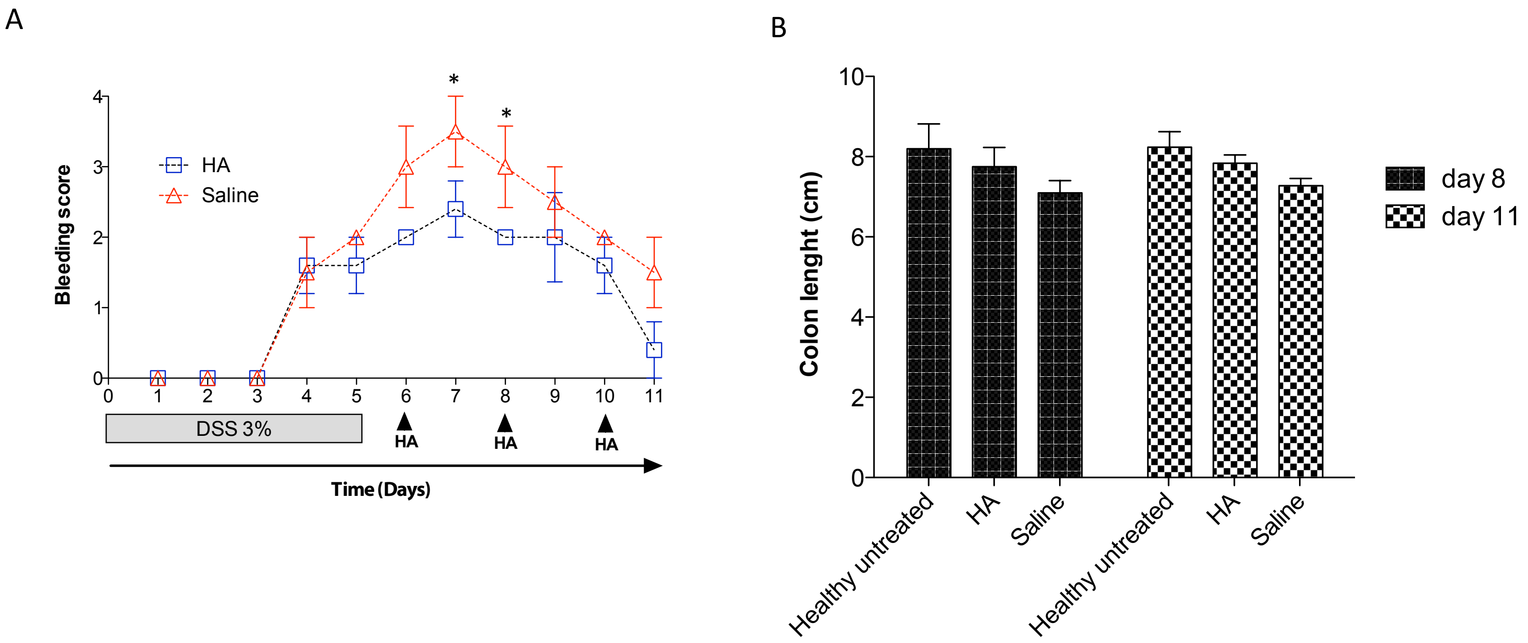

Figure S1. Clinical parameters recorded during the recovery phase in colitic mice after the administration of hyaluronan

Acute colitis was induced in C57BL/6N wild type mice by 3% DSS administration in drinking water *ad libitum* for 5 days. After this time DSS was replaced with regular water and starting on day 6 mice received every other day local applications of high molecular weight hyaluronan (HA, 1mg/mL) or saline via enema. **A** the bleeding score monitored over the entire experiment. **B** Colon length was measured at the end of the experiment (day 11).

Data represent means ± SEM (n= 5 /group). *P<0.05 compared to control group by one-way-ANOVA.

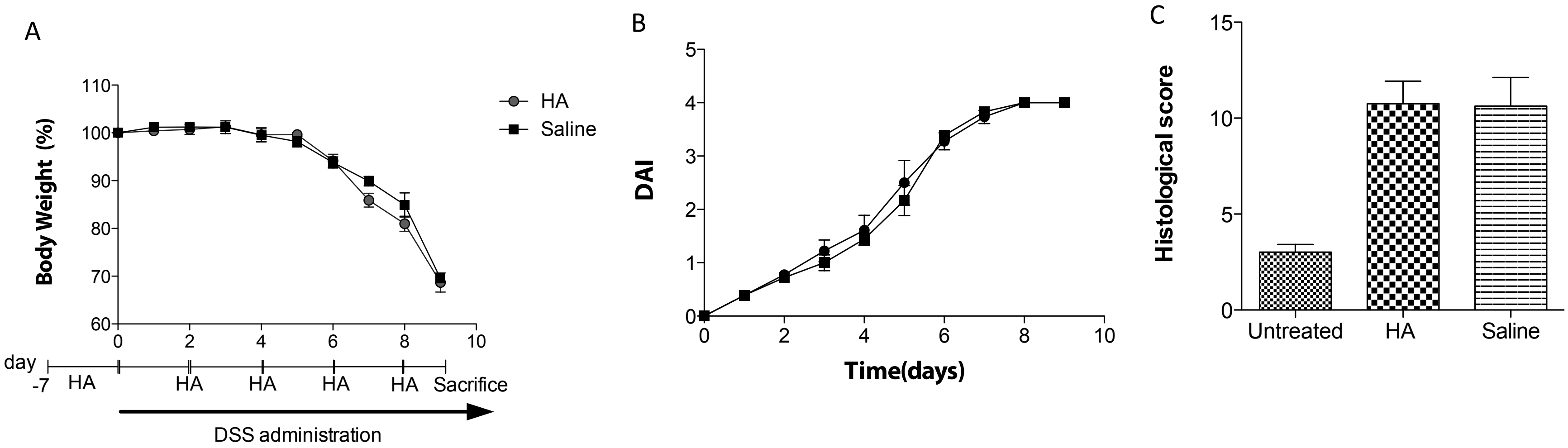

Figure S2. Lack of effects in preventing acute colitis in wild type mice receiving a pre-treatment with Hyaluronan.

C57BL/6N wild type mice received high molecular weight hyaluronan (HA, 1mg/mL) or saline via enema every other day during the course of experiment, starting 7 days before the administration of 3% DSS, which was given to mice via the drinking water for nine consecutive days to induce acute colitis. At day nine after the start of DSS treatment, all mice were sacrificed. **A-B**. Body weight and disease activity index (DAI) monitored over 9 days of DSS-induced colitis. C. Representative histological histograms of quantitative analysis of mucosal damage in all groups of mice. Data represent means ± SEM (n= 5 / group). **P*<0.05 compared to control group by one-way-ANOVA.

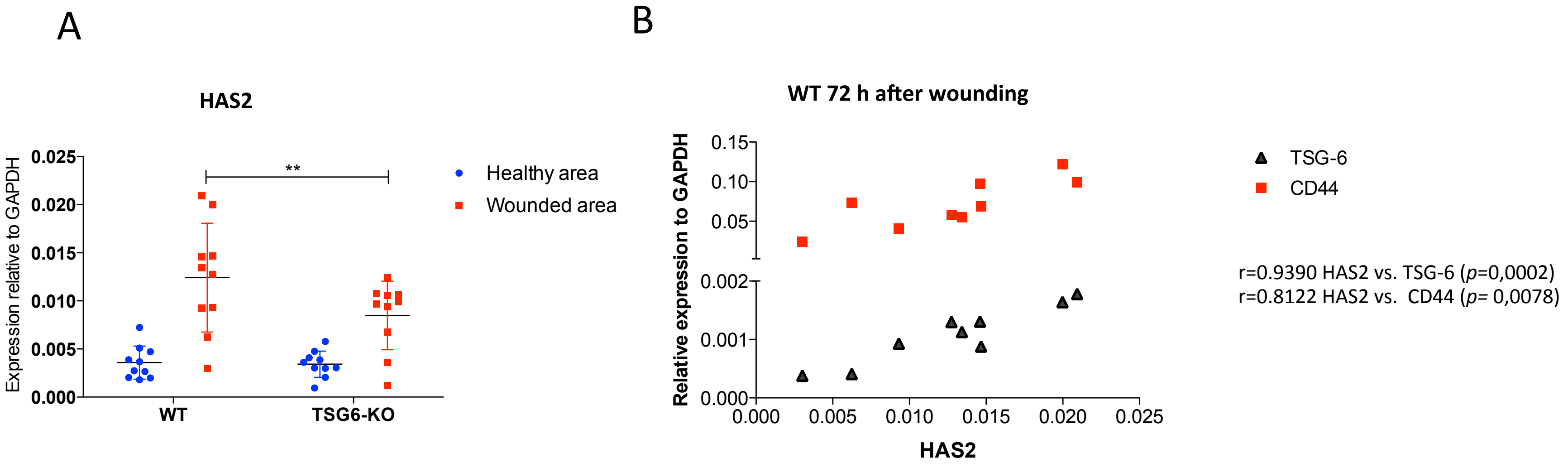

Figure S3. Analysis of mRNA levels of Hyaluronan-TSG-6/CD44 axis in wild type and TSG-6 deficient mice upon mucosal wounding .

A Gene expression of Hyaluronan Synthase 2 (HAS2) in mucosal wound of TSG-6 KO and wild type (WT) mice. A mucosal wound was created by taking a discrete biopsy from the distal colon of mice. After 72 hours, the wounded area was collected and analysed.

B Pearson correlation coefficient (r) between HAS2 vs. CD44 or vs TSG-6 gene expression in the wounded area of 9 WT mice. For all qRT-PCR results, data reflect mean ± SEM from 2 independent experiments; the results were normalized to glyceraldehyde-3-phosphate dehydrogenase (GAPDH) mRNA. **p<0.01 by one-way ANOVA. (n=9-10 animals/group)
